# Pretreatment with probiotics *Enterococcus faecium* NCIMB 11181 attenuated *Salmonella* Typhimurium-induced gut injury through modulating intestinal microbiome and immune responses with barrier function in broiler chickens

**DOI:** 10.1186/s40104-022-00765-5

**Published:** 2022-10-12

**Authors:** Yujing Shao, Wenrui Zhen, Fangshen Guo, Zeqiong Hu, Kaichen Zhang, Linhua Kong, Yuming Guo, Zhong Wang

**Affiliations:** 1 College of Biology, China Agricultural University, Beijing, China; 2 State Key Laboratory of Animal Nutrition, College of Animal Science and Technology, China Agricultural University, Beijing, China; 3 College of Animal Science and Technology, Henan University of Science and Technology, Province of Henan, Luoyang, China; 4 Tengzhou Heyi Food Co. Ltd, Zaozhuang, Shandong Province, China

**Keywords:** Broiler chickens, *Enterococcus faecium*, Gut health, *Salmonella* Typhimurium

## Abstract

**Background:**

Preventing *Salmonella* infection and colonization in young birds is key to improving poultry gut health and reducing *Salmonella* contamination of poultry products and decreasing salmonellosis for human consumption (poultry meat and eggs). Probiotics can improve poultry health. The present study was conducted to investigate the impact of a probiotics, *Enterococcus faecium* NCIMB 11181 (*E. faecium* NCIMB 11181) on the intestinal mucosal immune responses, microbiome and barrier function in the presence or absence of *Salmonella* Typhimurium (*S.* Typhimurium, ST) infection.

**Methods:**

Two hundred and forty 1-day-old *Salmonella*-free male broiler chickens (Arbor Acres AA^+^) were randomly allocated to four groups with 6 replicate cages of 10 birds each. The four experimental groups were follows: (1) negative control (NC), (2) *S.* Typhimurium, challenged positive control (PC), (3) the *E. faecium* NCIMB 11181-treated group (EF), (4) the *E. faecium* NCIMB 11181-treated and *S.* Typhimurium-challenged group (PEF).

**Results:**

Results indicated that, although continuous feeding *E. faecium* NCIMB 11181 did not obviously alleviate growth depression caused by *S.* Typhimurium challenge (*P* > 0.05), *E. faecium* NCIMB 11181 addition significantly blocked *Salmonella* intestinal colonization and translocation (*P* < 0.05). Moreover, supplemental *E. faecium* NCIMB 11181 to the infected chickens remarkably attenuated gut morphological structure damage and intestinal cell apoptosis induced by *S.* Typhimurium infection, as evidenced by increasing gut villous height and reducing intestinal TUNEL-positive cell numbers (*P* < 0.05). Also, *E. faecium* NCIMB 11181 administration notably promoting the production of anti-*Salmonella* antibodies in intestinal mucosa and serum of the infected birds (*P* < 0.05). Additionally, 16S rRNA sequencing analysis revealed that *E. faecium* NCIMB 11181 supplementation ameliorated *S.* Typhimurium infection-induced gut microbial dysbiosis by enriching *Lachnospiracease* and *Alistipes* levels, and suppressing *Barnesiella* abundance. Predicted function analysis indicated that the functional genes of cecal microbiome involved in C5-branched dibasic acid metabolism; valine, leucine and isoleucine biosynthesis; glycerolipid metabolism and lysine biosynthesis were enriched in the infected chickens given *E. faecium* NCIMB 11181. While alanine, asparate and glutamate metabolism; MAPK signal pathway-yeast; ubiquine and other terpenoid-quinore biosynthesis, protein processing in endoplasmic reticulum; as well as glutathione metabolism were suppressed by *E. faecium* NCIMB 11181 addition.

**Conclusion:**

Collectively, our data suggested that dietary *E. faecium* NCIBM 11181 supplementation could ameliorate *S.* Typhimurium infection-induced gut injury in broiler chickens. Our findings also suggest that *E. faecium* NCIMB 11181 may serve as an effective non-antibiotic feed additive for improving gut health and controlling *Salmonella* infection in broiler chickens.

## Background

*Salmonella enterica* var. Typhimurium (*S.* Typhimurium, ST), a rod-shaped, flagellated, aerobic and Gram-negative intracellular pathogen, which is one of the most prevalent serotypes of *Salmonella* in broiler chickens, and cause gastroenteritis in the human by entering the human food chain through animal products, particularly raw poultry products [1, 2]. *S.* Typhimurium infection in chicks younger than 2 weeks old results in poor growth rate, severe enteric and systemic disease with a high mortality rate along with persistent *Salmonella* infection in tolerant chickens. While *S.* Typhimurium infection in older chickens results in asymptomatic cecal colonization and persistent shedding of the organisms in feces, resulting in *Salmonella* contamination of poultry products, vertical transmission to offspring along with a cumulative economic loss [3–6]. Prolonged persistent infection with *S.* Typhimurium in the gut of chickens throughout their lifespan could not only alter the development of gut microbiota and have detrimental effect on the overall gut health of the chicken host, especially exposed to stress stimulation or other pathogens challenge. Therefore, reducing *S.* Typhimurium in the intestinal tract of chickens not only could reduce morbidity and mortality caused by *Salmonella* infection in young chicks, but also reduce *Salmonella* prevalence in poultry along with decrease contamination of poultry products and salmonellosis in humans.

Various preventive strategies, which include cleaning up the breeding herbs, general strict hygiene and biosecurity measures in the farm, vaccination, genetic selection of chicken lines with improved immunity, antibiotics, as well as supplementation with feed additives, such as organic acids, essential oils, bacteriophages, prebiotics, probiotics and etc., have been made by farmers to prevent *Salmonella* infection in poultry [5, 7]. Among above these measures, probiotics were considered as one of the most safest and effective measures employed in controlling *Salmonella* infections in poultry, because plenty of studies have demonstrated that probiotics could confer the health benefit on the host when administered in adequate amounts in chickens [8–11].

*Enterococcus faecium (E. faecium)* is a lactic acid bacterium and normal inhabitant in the gut. Although some strains of *Enterococcus* are pathogenic to human or animals, other strains such as *Enterococcus faecium* EF1, NCIMB 11181, NCIMB 10415, *E. faecium* SF68 and *E. faecium* M-74 are nonpathogenic and often used as commercial probiotics in medicine, food and animal feed, because of their resistance to low pH and bile salts, and encountered in digestion and produced enterococins [12–14]. Previous studies have demonstrated that feed or drinking water supplementation with *E*. *faecium* strain facilitates systemic and intestinal local mucosal immune responses [15–18], enhances disease resistance to pathogenic infection, partially prevents or treats diarrhea in pigs [19–23]. Furthermore, the addition of *E. faecium* to pig directly or indirectly modifies intestinal bacterial communities by increasing the prevalence of beneficial bacteria and reducing pathogenic bacteria load and/or increases growth performances [24–27]. Results from poultry experiments have revealed that supplementation of the diet with *E. faecium* strain improves growth performance, immune function, eggshell quality and modulates intestinal microflora composition [28–30]. Moreover, probiotics *E. faecium* supplementation has been reported to regulate intestinal mucosal immune responses and enhance chicken resistance to intestinal pathogen infection, such as *Salmonella* [31–34], *Escherichia coli (E. coli)* [35]. Indeed, probiotic strains differ regarding the properties and clinical effects that they elicit even if the strains belong to the same bacterial species. *E. faecium* strain NCIMB 11181 is currently authorized by the EFSA Panel on additives and products or substances used in animal feed as a supplement for fattening and improving the performance of animals. This strain has been shown to effectively increase daily weight gain, improve feed conversion and gut microbiota composition, together with enhance gut health in pigs [36]. In addition, our previous study had demonstrated that dietary *E. faecium* NCIMB 11181 addition could improve growth performance, and enhance cellular and humoral immunity of broiler chickens reared under non-challenged conditions [37]. We also found that pre-administration of *E. faecium* NCIMB 11181 could ameliorate necrotic enteritis-induced intestinal barrier injury in broilers [38], improve growth and reduced the death rate together with maintaining the intestinal integrity in *Escherichia coli* O78-challenged broiler chickens [39]. Some probiotics have been shown to be useful as antibiotics alternative for the control of some subclinical infections in poultry. Nevertheless, it is unknown whether dietary *E. faecium* NCIMB 11181 addition could be helpful for protecting intestinal health in broiler chickens infected with *S.* Typhimurium. Therefore, this study was conducted to investigate the effects of *E. faecium* NCIMB 11181 addition on *Salmonella* colonization and invasion, development of intestinal pathological lesions, intestinal immune response, together with intestinal barrier function in broiler chickens challenged with *S.* Typhimurium. In addition, we further assess the shifts in intestinal microbial community structure induced by dietary treatment and/or ST challenge to explain the possible protective effects of *E. faecium* NCIMB 11181 addition on broilers infected with ST.

## Materials and methodsf

### Animal ethics statement

Animal experiment was reviewed and approved by the Animal Care and Use Committee of China Agricultural University (Beijing, P. R. China).

### Experimental design, birds, diets and animal management

Two hundred and forty (*n* = 240) 1-day-old *Salmonella*-free male broiler chickens (Arbor Acres AA^+^) were purchased from a local supplier (Beijing Arbor Acres Poultry Breeding Company, Beijing, China). Birds were used to evaluate the protective efficacy of *E. faecium* NCIMB 11181 feed supplementation against ST infection. Meconium from each individual chicken was collected and checked it for *Salmonella* negativity using the plating method. Samples were pre-enriched with tetrathionate broth (CM 203–01, Beijing Land Bridge Technology Co., Ltd., Beijing, China) at 37 °C for 24 h, and then streaked on bismuth sulfite agar (CM 207, Beijing Land Bridge Technology Co., Ltd., Beijing, China) to confirm that the chicks were free of *Salmonella.* Subsequently the chicks were randomly divided into four experimental groups. These 240 two-day-old *Salmonella*-negative chickens was randomly assigned into four groups including: no additive and no challenge with *S.* Typhimurium (negative control, NC); no additive but challenged with *S.* Typhimurium (positive control, PC); *E. faecium*-supplemented but uninfected (EF); *E. faecium*-supplemented and infected with *S.* Typhimurium (PEF). Each group contained six replicate pens with 10 birds per pen and fed a balanced, un-medicated corn and soybean meal-based pelleted diet that contained either 0 or 200 mg/kg *E. faecium* NCIMB 11181, (viable count ≥ 2 × 10^9^ CFU/g; manufactured by Probiotics International Ltd. Co., UK)*.* To avoid cross-contamination, all uninfected birds were reared in one clean separate room, whereas all infected birds were housed in another room under the same environmental conditions. Antibiotic-free and coccidiostat-free corn-soybean meal-based pelleted diets were formulated to meet or exceed National Research Council (1994) requirements [40] and tested for the presence of *Salmonella.* The composition and nutrient levels of the basal diet is presented in Table 1. The experimental diet was formulated by mixing the basal diet with 200 g of *E. faecium* (2 × 10^9^ CFU/g of the product) to reach 4 × 10^8^ CFU/kg of diet. The feed samples were taken and the *E. faecium* number was counted by *Enterococcus faecium* agar (bile aesculin azide agar, HB0133-3) to ensure the probiotic dosages were performed correctly.

**Table 1:** Composition and nutrient levels of the experimental basal diet (as-fed basis unless stated otherwise, %)

Items	Content	Items	Content
Ingredients, %		**Calculated Nutrient levels**^**c**^	
Corn (CP 8.0%)	61.00	Metabolizable energy, kcal/kg	2.9072
Soybean meal (CP 43.0%)	33.00	Crude protein, %	19.24
Soybean oil	2.00	Total calcium, %	0.97
Limestone-calcium carbonate	1.21	Available phosphorus, %	0.43
Calcium hydrogen phosphate	1.83	Lysine, %	1.03
Sodium chloride	0.30	Methionine, %	0.41
*DL*-Methionine (98%)	0.10		
*L*-Lysine HCL (78%)	0.10		
Vitamin premix^a^	0.03		
Mineral premix^b^	0.20		
Choline chloride (50%)	0.20		
Ethoxyquin (33%)	0.03		
Total, %	100.00		

^a^Vitamin premix provided the following per kg of diets: vitamin A (retinylacetate), 12,500 IU; vitamin D_3 _(cholecalciferol), 2500 IU; vitamin E (*DL*-a-tocopherol acetate), 18.75 mg; vitamin K_3_ (menadione sodium bisulfate), 2.65 mg; VB_1_, 2 mg; VB_2_, 6 mg; VB_6_, 6 mg; vitamin B_12_ (cyanocobalamin), 0.025 mg; biotin, 0.0325 mg; folic acid, 1.25 mg; pantothenic acid, 1.25 mg; nicotinic acid, 50 mg

^b^Mineral premix provided per kilogram of complete diet: Cu (as copper sulfate) 8 mg, Zn (as zinc sulfate) 75 mg, Fe (as ferrous sulfate) 80 mg, Mn (as manganese sulfate) 100 mg, Se (as sodium selenite) 0.15 mg, I (as potassium iodide) 0.35 mg

^c^Calculated value based on the analyzed data of experimental diets

The chicks were reared on net floor cages in a closed and ventilated house. Each pen had a floor space of 7200 (120 × 60) cm^2^ and was equipped with a separate feeding trough. Water was supplied through nipple drinkers. Water and feed were provided ad libitum. In accordance with the AA^+^ Broiler Management Guide, all chicks received continuous light for the first 24 h, and were then maintained under a 23-h light/1-h dark cycle for the remainder of the study. The room temperature was maintained at 33–34 °C on the first 3 days, and then gradually decreased by 2 °C/week until a final room temperature of 22–24 °C of reached. The relative humidity was kept at 60%–70% during the first week and then 50%–60% thereafter.

### *Salmonella* Typhimurium challenge

The *Salmonella enterica* serovar Typhimurium CVCC 2232 was obtained from the China Veterinary Culture Collection Center (Beijing, China). The frozen culture was recovered by using sterile buffered peptone water (CM201, BPW, Beijing Land Bridge Technology Co., Ltd, Beijing, China). ST pre-culture was transferred to 100 mL of tryptone soy broth (CM201, TSB, Beijing Land Bridge Technology Co., Ltd, Beijing, China) and incubated at 37 °C with orbital shaking for 16 to 18 h. The concentration of viable ST in the culture was counted on bismuth sulfite agar (CM207, BS, Beijing Land Bridge Technology Co., Ltd, Beijing, China) at 37 °C for 24 h and the stock culture was adjusted to a final concentration of 1 × 10^9^ CFU/mL ST. At 10 and 11 days of age, birds in the ST-challenged groups were inoculated with 1 mL of bacterial suspension containing approximately 1 × 10^9^ colony forming units (CFU) of ST suspension by gavage. Unchallenged groups received 1.0 mL of PBS without ST on the same date. Feed was withdrawn from all birds 10 h before challenge.

### Measurement of growth performance

Body weight of broiler in each replicate was measured individually at 1, 10 and 18 days of bird age. Average body weight (ABW) and average body weight gain (BWG) were calculated during different periods (during d 1 to 10, and d 11 to 18).

### Samples collection

On d 7 after the *S.* Typhimurium challenge, all birds from each group were euthanized via cervical dislocation, and livers, spleen and left cecal contents of each bird were aseptically harvested and assessed for ST content as soon as possible. At the same time, blood, ileum and cecal contents from only 8 chickens in each treatment group were collected for subsequent analysis. Details are as follows, blood were collected for serum anti-*Salmonella* specific IgG analysis; the right cecal contents were aseptically collected, snap-frozen in liquid nitrogen and then stored at –80 °C for intestinal microbial 16S rDNA-based analysis. Proximal ileum segments were flushed with 0.05 mol/L PBS, pH 7.2 and fixed in 4% (w/v) polyoxymethylene solution for histological and immunohistochemistry examination. Distal ileum parts were collected, washed for 2 times with ice-cold PBS, and then snap-frozen in liquid nitrogen for mRNA determination. Ileal mucosa from each bird was collected and homogenized in ice-cold PBS (pH 7.2), and centrifuged, then the supernatant was collected and stored at –20 °C for anti-*Salmonella* specific IgA analysis.

### Detection of *Salmonella* in cecal contents and internal organs

*Salmonella* numbers in cecal contents and internal organ were determined as described previously [5]. Briefly, samples of liver, spleen and cecal contents were weighed, homogenized in BPW (10% w/v suspensions) for 1 min using a Stomacher respectively and serially diluted tenfold (1:10) with sterile PBS to appropriate levels for *Salmonella* numeration on xylose lysine tergitol 4 agar (CM219-07, XLT4, Beijing Land Bridge Technology Co., Ltd, Beijing, China) plates containing 100 μg/mL nalidixic acid. The number of black bacterial colonies was determined counted on XLT4 agar plates after incubation for 24 h at 37 °C and expressed as mean ± standard error of the mean log_10_ CFU/g feces or tissues. Samples that were positive only after enrichment with tetrathionate broth (CM203, TTB, Beijing Land Bridge Technology Co., Ltd, Beijing, China) and then streaked into XLT4 agar plated containing 100 μg/mL nalidixic acid solution were counted as 1 CFU/g, and samples that yielded no *Salmonella* growth after enrichment were counted as 0 CFU/g. A *Salmonella*-positive bird was defined based on recovery of *Salmonella* from any of the internal organs (liver, spleen) studied in an assay. The percent efficacy of protection for a particular group was calculated based on the number of *Salmonella*-positive birds out of the total number of birds in a group.

### Intestinal histology and immunohistochemical staining analysis

Ileal samples were collected and fixed in 4% paraformaldehyde after postmortem examination, and then processed, trimmed, and embedded in paraffin by routine methods. The serial paraffin Sections (5 μm) were prepared and stained with hematoxylin–eosin (HE) for histological (Villous height (VH) and crypt depth (CD), magnification × 40) [5]. In addition, HE-stained 5-µm-thick sections was determined intestinal inflammation or pathological scores as previously described [4] using a light microscope (Leica model DMi8, Leica, Wetzlar, Germany) at magnification of × 200. All scores were obtained in a blinded fashion by two independent investigators. The terminal-deoxynucleoitidyl transferase mediated nick end labeling (TUNEL) assay of ileum-tissue sections was performed by using immunostaining following the same procedure as described in our previous study [39]. The integral optical density (IOD) of TUNEL-positive cells in the ileum was assessed by a digital microscope and camera system (Nikon DS-Ri1, Japan).

### Quantitative real-time PCR

Total RNA isolation, reverse transcription, and real-time PCR were carried out as previously described [39]. The primers for real-time PCR are listed in Table 2. The efficiency of all tested genes was between 90% and 110%. All the tissue samples for the cDNA synthesis and in the following PCR amplifications were run in triplicate. Gene expression for immune-related genes (*TLR4, MyD88, NF-κB, IFN-γ, IL-1β, IL-6, IL-8, TNF-SF15, TGF-β4, PIgR, A20*, *Tollip* and *PI3K*), tight junction proteins-related genes (*Claudin-1, Occludin, ZO-1, ZO-2* and *MLCK*) was analyzed using glyceraldehyde-3-phosphate dehydrogenase (*GAPDH*) as an endogenous control. The method of 2^−ΔΔCt^ was used to analyze the real-time PCR data [41] and results were expressed as the fold change relative to the average value of the negative control group (the non-treated and non-challenged control).

**Table 2:** Sequences of the oligonucleotide primers used for quantitative real-time PCR for immune-related genes expreesion^a^

Genes	Forward primer sequence (5′ → 3′)	Reverse primer sequence (5′ → 3′)	GenBank accession No	PCR size, bp
*TLR4*	F:CCACTATTCGGTTGGTGGAC	R:ACAGCTTCTCAGCAGGCAAT	NM_001030693.1	120
*MyD88*	F:TGCAAGACCATGAAGAACGA	R:TCACGGCAGCAAGAGAGATT	NM_001030962.3	123
*NF-kB*	F:TGGAGAAGGCTATGCAGCTT	R:CATCCTGGACAGCAGTGAGA	NM_205134.1	117
*IL-1β*	F:TCATCTTCTACCGCCTGGAC	R:GTAGGTGGCGATGTTGACCT	NM_204524.1	149
*IL-6*	F:GATCCGGCAGATGGTGATAA	R:AGGATGAGGTGCATGGTGAT	NM_204628.1	126
*IL-8*	F-GGCTTGCTAGGGGAAATGA	R-AGCTGACTCTGACTAGGAAACTGT	NM_205498.1	200
*TNF-SF15*	F-CCCCTACCCTGTCCCACAA	R-TGAGTACTGCGGAGGGTTCAT	NM_204267.1	67
*IFN-γ*	F:CTTCCTGATGGCGTGAAGA	R:GAGGATCCACCAGCTTCTGT	NM_205149.1	127
*TGF-β4*	F:AGAGCATTGCCAAGAAGCAC	R:GCAGTAGTCGGTGTCGAGGT	NM_001318456.1	119
*A20*	F:GAGAACGCAGAGCCTACACC	R:CCAACCTTCTTCCTGCACAT	NM_001277522.1	95
*Tollip*	F:CATGGTACCTGTGGCAATACC	R:GCACTGAGCGGATTACTTCC	NM_001006471	122
*PI3K*	F:AACATCTGGCAAAACCAAGG	R:CTGCAATGCTCCCTTTAAGC	NM001004410	150
*PIgR*	F:ATGAAGCAGAGCCAGGAGAC	R:GAGTAGGCGAGGTCAGCATC	NM001044644.1	128
*MLCK*	F:TTGACATGGAGGTTGTGGAA	R:GAAGTGACGGGACTCCTTGA	NM_001322361.1	119
*Claudin1*	F:AAGTGCATGGAGGATGACCA	R:GCCACTCTGTTGCCATACCA	NM_001013611.2	119
*Occludin*	F:AGTTCGACACCGACCTGAAG	R:TCCTGGTATTGAGGGCTGTC	NM_205128.1	124
*ZO-1*	F:ACAGCTCATCACAGCCTCCT	R:TGAAGGGCTTACAGGAATGG	XM_015278981.1	125
*ZO-2*	F:CACCACCACCTGTTTCTGTG	R:TTCACTCCCTTCCTCTTCCA	NM_204918.1	119

^a^Primers were designed and synthesized by Sango Biotech (Shanghai, China) Co., Ltd. *F:* forward, *R*: reverse

*TLR* Toll-like receptor, *MyD88* myeloid differential protein-88, *TRAF-6* TNF receptor-associated factor 6, *NF-κB* nuclear factor kappa-light-chain-enhancer of activated B cells, *TNFSF15* tumor necrosis factor superfamily member 15, *IL* interleukin, *IFN-γ* interferon γ, *Tollip* Toll-interacting protein, *PI3K* phosphatidylinositol 3-kinase, *A20* protein A20, *SOCS* suppressor of cytokine signaling, *ZO-1* zonula occludens-1, *EGFR* epidermal growth factor receptor, *GLP-2* lucagon-like peptide-2, *IGF-2* insulin-like growth factor-2, *TGF- β3 *transforming growth factor beta 3

### Measurement of anti-*Salmonella* specific antibody in the intestine and serum

Briefly, ST CVCC 2232 (10^8^ CFU/mL) cells were washed 3 times and lysed by an ultrasonic processor 250 (USA) at 85 W and 30-s intervals on ice for 5 min. The lysed cells were centrifuged at 10,000 × *g* for 10 min, and the resultant supernatant was collected and stored at –70 °C until use. Flat-bottomed 96-well ELISA microplates (Corning, NY, USA) were coated with 100 μL of 20 μg/mL of the antigen diluted in 0.1 mol/L carbonate-bicarbonate buffer (15 mmol/L Na_2_CO_3_, 35 mmol/L NaHCO_3_, 0.3 mmol/L NaN_3_) for the measurement of anti–ST specific IgG in the serum and specific IgA in intestinal mucosa homogenate, respectively, using an indirect enzyme-linked immunosorbent assay (ELISA) as described previously [42]. Serum samples were diluted 1:100 and intestinal wash samples were diluted 1:5 in PBST with 1% bovine serum albumin (BSA). Absorbance values (optical density, OD) were read at 450 nm using an automatic ELISA reader (Bio-Tek EL311sx autoreader, Bio-Tek, USA). Each serum sample or intestinal sample was tested in duplicate.

### Microbial DNA extraction, 16S rRNA amplification, sequencing and bioinformatic analysis

Microbial DNA was extracted from cecum contents of broilers using QIAamp DNA Stool Mini Kits protocol (Qiagen Inc, Germany) according to the manufacturers’ protocol. The quality and quantity of DNA samples were determined using a Nanodrop ND-1000, and agarose gel electrophoresis was used to confirm the absence of degradation. The bacterial 16S rRNA gene V3-V4 region was amplified using the KAPA HiFi Hotstart ReadyMix PCR kit (Kapa Biosystems, USA) and primers F341 and R806 (F341: 5′-ACTCCTACGGGRSGCAGCAG-3′, R806: 5′-GGACTACVVGGGTATCTAATC-3′). 16S rRNA gene sequencing was performed using the Illumina HiSeq PE250 sequencing platform (Illumina, San Diego, United States) at Biomarker Technology Co., Ltd. (Beijing, China). FLASH (FLASH: fast length adjustment of short reads to improve genome assemblies) was applied for the assembly of resulting 300-bp paired-end reads [43]. Additional sequence read processing, which included quality filtering based on a quality score > 25 and removal of mismatched barcodes and sequences below length thresholds, was performed within QIIME (version 1.9.1) [44]. USEARCH (version 7, 64-bit) was further utilized for denoising and chimera detection [45]. All of the effective reads from each sample were clustered into operational taxonomic units (OTUs) based on a 97% sequence similarity identified by UCLUST in QIIME (version 1.9.1 [44]. Taxonomic classification at different taxonomic levels of OUT sequences were performed by comparing sequences to the GreenGene v13.8 database [46]. Shannon and Simpson indices, Chao1 and ACE estimators were included in α-diversity analysis by using the MOTHUR v1.31.2 [47]. The principal coordinates analysis (PCoA) and partial least squares discriminant analysis (PLS-DA) plots based on weighted and unweighted Unifrac distance matrices were used to estimate pairwise distances among samples and to establish β-diversity. Analysis of similarities (ANOSIM) with 999 permutations was used to detect statistical significances between microbial communities in different groups. This test measures a value of *R*, normally scaled from 0 to 1, which is based on the average rank similarity among groups and replicates within each group [48]. *R* = 0 indicates that two groups are similar, whereas *R* = 1 shows a perfect separation between groups. Linear discriminant analysis (LDA) combined effect size measurements (LEfSe) and non-parametric *t*-test (with Metastats software) were further employed to identify the biological differences in the microbial composition among groups [49]. LDA was performed from the phylum to genus level, and LDA scores ≥ 4.0, and *P*-values < 0.05 were selected for plotting and further analysis.

### Functional analysis of the gut microbiota

Metagenome functional content from high-quality 16S rDNA was predicted using the phylogenetic investigation of communities by reconstruction of unobserved states (PICRUSt) software [50], based on the Kyoto Encyclopedia of Genes and Genomes (KEGG) Orthology database. Significance analysis was performed by two-way ANOVA using GraphPad Prism 5 (GraphPad Software, San Diego, CA, USA). Data were then analyzed with Statistical Analysis of Taxonomic and Functional Profiles (STAMP) version 2.1.3 [51]. Differentially represented functional pathways (level 2 in hierarchy, representing KEGG pathways) between the two conditions (presented in extended error bar plots) were analyzed with two-sided Welch’s *t*-test on every pair of means where *P* < 0.05 was considered significant. Confidence intervals of 95% were obtained by inverting the Welch’s tests.

### Statistical analysis

The growth performance data, intestinal structure data, intestinal apoptosis index, intestinal and internal organs salmonella numbers, antibody levels and gene expression data were subjected to two-way ANOVA by using the GLM procedure of the SPSS, version 18.0 (SPSS Inc., Chicago, IL, USA). The model included the main effect of probiotics treatments, *Salmonella* challenge and their interaction. The relative abundance of microorganisms obtained from 16S rRNA sequencing was analyzed using the Kruskal–Wallis rank sum test to compare the difference between the comparison groups. Significance was set at *P* < 0.05, and a trend towards significance at *P* < 0.10 was seen. Data in the tables were expressed as means and pooled SEM.

## Results

### Growth performance

Data on growth performance including body weight (BW), body weight gain (BWG) of different phase are shown in Table 3. *S.* Typhimurium challenge resulted in a significant reduce in BW (at d 21) and BWG (during d 12 to 21) compared with the non-challenged birds (*P* < 0.05), while the addition of *E. faecium* NCIMB 11181 in feed had no remarkable influence on growth performance (BW and BWG) in broiler chickens irrespective of ST challenge (*P* > 0.05). No significant difference for mortality rate was observed in all groups during the experimental period (*P* > 0.05).

**Table 3:** Effect of dietary *Enterococcus faecium* NCIMB 11181 supplementation on broiler chicken performance challenged with *Salmonella* Typhimurium

Items	ST^1^	Body weight, g/bird		Body weight gain, g/bird	
		d 10	d 18	d 1–10	d 11–18
Dosage, mg/kg					
0	–	182	500	144	318
200	–	193	530	154	337
0	+	183	482	144	298
200	+	176	458	138	281
SEM^2^		2.78	9.87	2.73	7.60
Main factors					
0		183	491	144	308
200		185	494	146	309
Non-challenged		188	515^a^	149	327^a^
Challenged		180	470^b^	141	290^b^
Main factors and Interaction (*P* value)^3^					
*Enterococcus faecium*		0.697	0.819	0.694	0.905
Challenged		0.161	0.011	0.176	0.006
*Enterococcus faecium* × Challenged		0.108	0.082	0.136	0.106

^1^Challenged with *Salmonella* Typhimurium; –, without ST challenged; +, with ST challenged

^2^SEM, standard error of the mean

^3^*P*-value represent the main effect of the diet, the main effect of ST challenged, and the interaction between the dietary treatments and ST challenged

^a,b^Means in the same column without common superscripts differ significantly (*P* < 0.05)

### Intestinal histopathological scores and intestinal cell apoptosis index

**Fig. 1:**
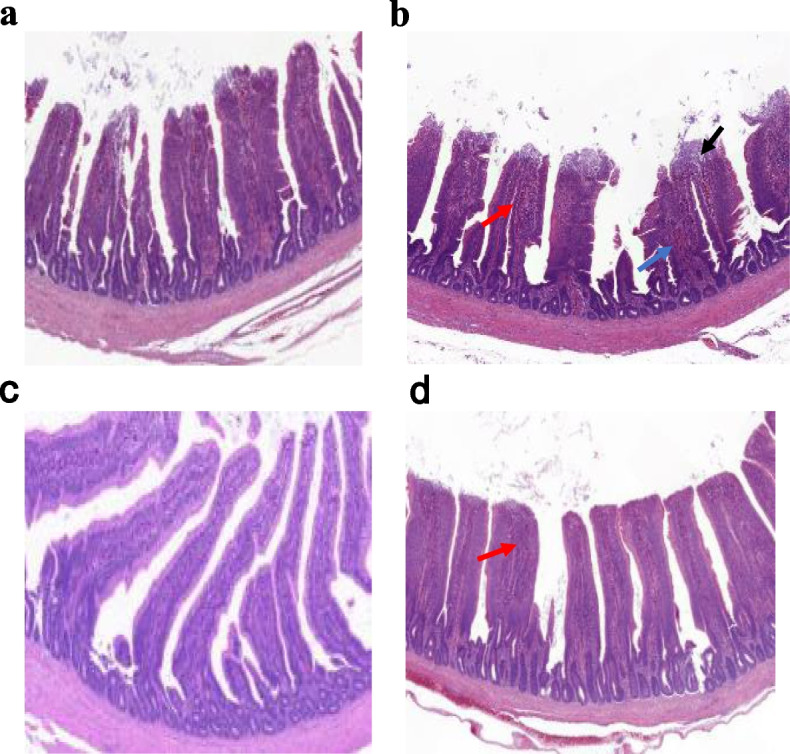
Histopathological changes in the ileum of the NC group (**a**), the PC group (**b**), the EF group (**c**), and the PEF group (**d**). The histological structure of chicken ileum was observed by H&E staining. Magnification was 40 × , and the scale bar was 20 μm. Red arrow, lymphocytes infiltration; blue arrow, hemorrhage; black arrow, defects of epithelium at the tip of villus. NC non-infected and untreated negative control, PC ST-infected positive control without probiotics addition, EF *E.* *faecium-*treated group without ST infection, PEF both *E. faecium*-treated and ST-infected group. ST *Salmonella* Typhimurium

**Fig. 2:**
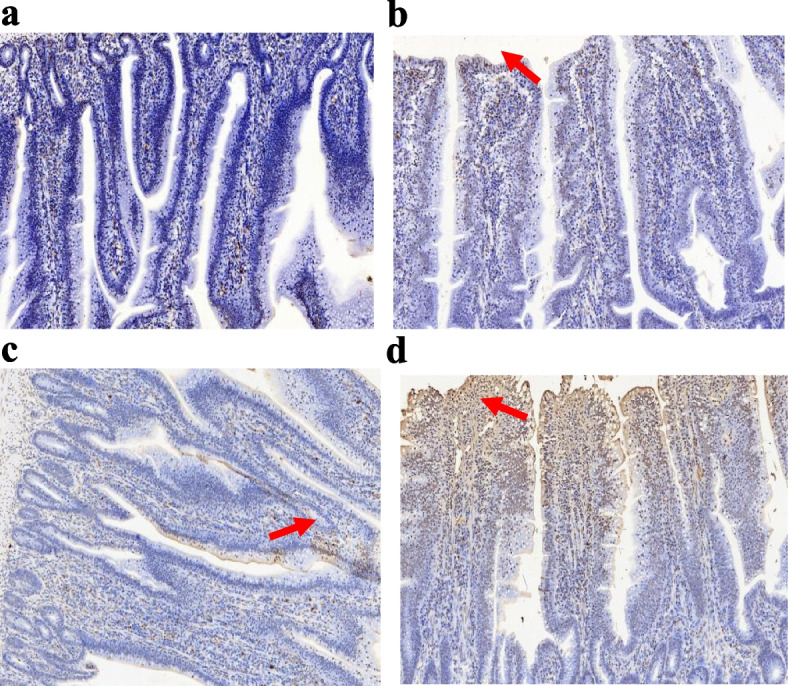
A TUNEL assay in the ileum sections after 7 days of *Salmonella* Typhimurium (ST) infection in broiler chickens from (**a**) the NC group, (**b**) the PC group, (*c*) the EF group, and (**d**) the PEF group. The blue color represents the live cells in the jejunal villus, and the brown color represents the apoptotic cells. The red arrow points out a typical TUNEL-positive cell. Magnification = 200 × . NC  non-infected and untreated negative control, PC ST-infected positive control without probiotics addition, EF  *E.* *faecium*-treated group without ST infection, PEF both *E.* *faecium-*treated and ST-infected group. ST *Salmonella* Typhimurium

A small number of inflammatory cells were found only in the ileum of the ST group, indicating a mild inflammatory reaction at 7 days post *S.* Typhimurium infection (dpi), while no obvious inflammatory cells infiltration were found in the other three groups (Fig. 1). Moreover, *S.* Typhimurium strongly increased the inflammation score, and TUNEL-positive cell numbers, significantly decreased villous height, and the ratio of villous height to crypt depth (V/C) (*P* < 0.05) as compared to the non-infected groups. *E. faecium* NCIBM 11181 pretreatment significantly decreased the inflammation score and TUNEL-positive cells content, moreover, promoted the growth of villous height irrespective of ST infection (*P* < 0.05), but the crypt depth and V/C was not affected by *E. faecium* NCIBM 11181 (*P* > 0.05) (Table 4; Fig. 2). However, no significant interaction effect was observed in intestinal histopathological scores, cell apoptosis rates at 7 dpi in the ileum among the four treatment groups (*P* > 0.05).

**Table 4:** Effect of dietary *Enterococcus faecium* NCIMB 11181 supplementation on intestinal morphology, histopathological scores and intestinal cells apoptosis index (Tunel) of broiler chickens challenged with *Salmonella* Typhimurium

Items	ST^1^	Villous height, μm	Crypt depth, μm	V/C	HE scores	Tunel
Dosage, mg/kg						
0	–	899.2	134.5	6.81	1.46	7767
200	–	957.7	145.4	6.59	1.00	3514
0	+	743.4	133.2	5.58	4.00	7538
200	+	832.2	142.6	5.85	3.40	5391
SEM^2^		43.27	5.92	0.293	0.334	1275.7
Main factors						
0		821.7^a^	133.9	6.18	2.81^a^	7652^a^
200		895.3^b^	144.0	6.22	2.09^b^	4452^b^
Non-challenged		928.3^a^	140.5	6.70^a^	1.24^b^	5641^b^
Challenged		788.7^b^	137.8	5.71^b^	3.70^a^	6464^a^
Main factors and Interaction (*P*-value)^3^						
* Enterococcus faecium*		0.042	0.498	0.537	0.047	0.016
Challenged		0.001	0.251	0.048	0.000	0.037
* Enterococcus faecium* × Challenged		0.298	0.497	0.264	0.392	0.057

^1^Challenged with *Salmonella* Typhimurium, –, without ST challenged, + , with ST challenged

^2^SEM, standard error of the mean

^3^*P*-value represent the main effect of the diet, the main effect of ST challenged, and the interaction between the dietary treatments and ST challenged

^a,b^Means in the same column without common superscripts differ significantly (*P* < 0.05)

### *Salmonella* numbers in cecal contents and internal organs

Bacterial translocation occurs when the barrier function of the intestine is impaired. Therefore, we checked the number of *Salmonella* colonies in the intestines and internal organs. All samples that were taken from uninfected control chicks were negative for *S.* Typhimurium. The efficacy of *E. faecium* NCIMB 11181 supplementation in reducing *Salmonella* colonization and invasion was evaluated by bacterial counting of the ST challenge strain in liver, spleen and cecal content of broiler chickens (Table 5). Table 5 shows that at 7 days post-challenge, *Salmonella* was detected in the liver, spleen and cecal content only after enrichment in all challenged groups (PC and PEF) with significant differences in *Salmonella* burden in the liver and cecum compared with un-challenged groups (*P* < 0.05). Challenged-birds fed diets with *E. faecium* NCIMB 11181 resulted in significant reduction in cecal *Salmonella* counts in comparison with the challenged but un-supplemented birds. *Salmonella* load in the liver of the *E. faecium* NCIMB 11181-treated birds tended to be lower than that of the single ST-challenged group, though not statistically significantly. After direct enrichment cultures, the number of *Salmonella* -positive birds (liver) was significantly lower in the *E. faecium* NCIMB 11181-treated birds (6/29) than in the single challenged control (14/29) at d 7 post-challenge, whereas no significant difference was found in the spleen. The results showed that compared with the ST group, the number of the liver and cecum in the ST-infected birds fed *E. faecium* NCIMB 11181 group was significantly lower in both liver tissue and cecum content, indicating that *E. faecium* NCIMB 11181 can reduce bacterial colonization and prevent bacterial translocation.

**Table 5:** Effect of dietary *Enterococcus faecium* NCIMB 11181 supplementation on the number of *Salmonella* in the liver, spleen and cecal contents (CFU/g) and the percentage of *Salmonella*-positive bird (liver, spleen) in *Salmonella*-challenged broilers chickens

Items	ST^1^	*Salmonella* recovery^5^				
Dosage, mg/kg		Liver		Spleen		Cecum
		CFU/g^4^	No. positive^6^	CFU/g^4^	No. positive^6^	
0	–	0.00	0/30	0.00	0/30	0.00^c^
200	–	0.00	0/30	0.00	0/30	0.00^c^
0	+	0.74	14/29	0.29	7/29	6.85^a^
200	+	0.29	6/29	0.22	5/29	5.32^b^
SEM^2^		0.125		0.089		0.146
Main factors						
0		0.37		0.14		3.43
200		0.14		0.11		2.66
Non-challenged		0.00^b^		0.00		0.00
Challenged		0.52^a^		0.25		6.09
Main factors and Interaction (*P-*value)^3^						
* Enterococcus faecium*		0.350		0.842		0.042
Challenged		0.038		0.171		< 0.001
* Enterococcus faecium* × Challenged		0.350		0.842		0.031

^1^Challenged with *Salmonella* Typhimurium, –, without ST challenged, + , with ST challenged

^2^SEM, standard error of the mean

^3^*P*-value represent the main effect of the diet, the main effect of ST challenged, and the interaction between the dietary treatments and ST challenged

^4^CFU/g recovered from tissues expressed in log10 values

^5^Challenge strain recovery by direct and enrichment cultures of liver, spleen, and cecum of birds

^6^Number of positive samples per total number of samples after bacterial recovery

^a,b^Mean ± SD in the same column without common superscripts differ significantly (*P* < 0.05)

### Ileum immune-related genes expression

To explain the anti-inflammatory action of *E. faecium* NCIMB 11181, the mRNA levels of TLR-mediated signal pathway molecules, i.e. *TLR4, MyD88, NF-κB, IL-1β, IL-6, IL-8, TNF-α, TGF-β4, IFN-γ, pIgR* and negative regulators *A20, Tollip* and *PI3K* in the chicken ileum mucosa were measured at 7 days post ST infection. Results showed that the expression levels of *MyD88, NF-κB, IFN-γ, pIgR* and negative regulators *Tollip* and *PI3K* in the chickens of the infected group were significantly upregulated (*P* < 0.05) than those of the non-challenged control groups (Table 6). ST infection also showed an increased trend for *TLR4* (*P* = 0.099) and *TNF-α* (*P* = 0.096) mRNA levels. While only *pIgR* and *Tollip* genes expression are remarkably up regulated by the addition of *E. faecium* NCIMB 11181 to the diets. Significant upregulation of *pIgR* and *Tollip* mRNA expression level in the ileum was observed in the *E. faecium* NCIMB 11181-treated groups compared with the non-supplemented birds. However, there was no significant cooperative effects on immune-related molecules expression between ST challenge and *E. faecium* NCIMB 11181 addition.

**Table 6:** Effect of dietary *Enterococcus faecium* NCIMB 11181 supplementation on immune-related gene expression in the ileum of broilers challenged with *Salmonella* Typhimurium

Items	ST^1^	*TLR4*	*MyD88*	*NF-κB*	*IL-1β*	*IL-6*	*IL-8*	*TNF-SF15*	*IFN-γ*	*TGF-β4*	*PlgR*	*A20*	*Tollip*	*PI3K*
Dosage, mg/kg														
0	–	1.03	1.07	1.02	1.07	1.12	1.08	1.02	1.02	1.02	1.04	1.01	1.03	1.02
200	–	1.00	0.94	1.09	1.38	0.75	1.93	1.01	1.52	0.80	1.28	1.08	1.21	1.16
0	+	1.33	1.23	1.19	1.85	1.14	1.96	1.13	2.61	0.77	1.60	1.11	1.26	1.56
200	+	1.31	1.47	1.33	2.03	0.82	1.88	1.50	2.20	0.76	2.39	1.05	1.65	1.44
SEM		0.086	0.074	0.047	0.214	0.149	0.287	0.090	0.184	0.084	0.125	0.032	0.070	0.083
Main factors^2^														
0		1.15	1.15	1.11	1.46	1.13	1.52	1.08	1.82	0.90	1.23^b^	1.06	1.15^b^	1.29
200		1.16	1.20	1.21	1.71	0.78	1.91	1.26	1.83	0.78	1.71^a^	1.06	1.43^a^	1.30
Non-challenged		1.02	1.01^b^	1.05^b^	1.23	0.94	1.50	1.02	1.27^b^	0.92	1.16^b^	1.04	1.12^b^	1.09^b^
Challenged		1.32	1.35^a^	1.26^a^	1.94	0.98	1.92	1.32	2.43^a^	0.77	1.99^a^	1.08	1.46^a^	1.50^a^
Main factors and Interaction (*P-v*alue)^3^														
* Enterococcus faecium*		0.910	0.699	0.237	0.573	0.279	0.523	0.296	0.883	0.516	0.049	0.954	0.019	0.950
Challenged		0.099	0.017	0.024	0.110	0.898	0.493	0.096	0.001	0.417	0.004	0.572	0.006	0.014
* Enterococcus faecium* × Challenged		0.975	0.170	0.714	0.882	0.940	0.447	0.282	0.116	0.552	0.520	0.320	0.339	0.426

^1^Challenged with *Salmonella* Typhimurium, –, without ST challenged, + , with ST challenged

^2^SEM, standard error of the mean

^3^*P*-value represent the main effect of the diet, the main effect of ST challenged, and the interaction between the dietary treatments and ST challenged

^a,b^Means in the same column without common superscripts differ significantly (*P* < 0.05)

### Gene expressions of intestinal tight junction

In order to investigate why the intestinal integrity was changed by *S.* Typhimurium and *E. faecium* NCIMB 11181, expression of selected *MLCK* and tight junction genes was measured by RT-PCR. As showed in Table 7, in comparison with the non-challenged group, *Salmonella* infection significantly decreased mRNA levels of tight junction *Claudin-1, Occludin*, *ZO-1* and *ZO-2* while increased the mRNA level of *MLCK* (*P* < 0.05). No significant difference in *MLCK, Claudin-1, Occludin, ZO-1* and *ZO-2* at the mRNA level was observed in the *E. faecium* NCIMB 11181 group compared with the un-supplemented group regardless of ST infection (*P* > 0.05). Moreover, there was no significant interactive effects on *MLCK* and tight junction expression at 7 d post-infection between ST challenge and *E. faecium* NCIMB 11181 supplementation.

**Table 7:** Effect of dietary *Enterococcus faecium* NCIMB 11181 supplementation on mRNA abundance of tight junction in the ileum of broiler chickens challenged with *Salmonella* Typhimurium

Items	ST^1^	*MLCK*	*Claudin-1*	*Occludin*	*ZO-1*	*ZO-2*
Dosage, mg/kg						
0	–	1.03	1.05	1.06	1.02	1.01
200	–	0.85	0.86	0.74	0.98	1.04
0	+	1.61	0.48	0.47	0.69	0.68
200	+	2.03	0.58	0.59	0.74	0.66
SEM^2^		0.153	0.070	0.074	0.052	0.054
Main factors						
0		1.30	0.79	0.79	0.87	0.86
200		1.44	0.73	0.68	0.86	0.85
Non-challenged		0.94^b^	0.95^a^	0.90^a^	1.00^a^	1.02^a^
Challenged		1.84^a^	0.53^b^	0.52^b^	0.72^b^	0.67^b^
Main factors and Interaction (*P* -value)^3^						
* Enterococcus faecium*		0.638	0.682	0.419	0.981	0.971
Challenged		0.002	0.001	0.007	0.005	0.000
* Enterococcus faecium* × Challenged		0.233	0.197	0.082	0.660	0.763

^1^Challenged with *Salmonella* Typhimurium, –, without ST challenged, + , with ST challenged

^2^SEM, standard error of the mean

^3^*P*-value represent the main effect of the diet, the main effect of ST challenged, and the interaction between the dietary treatments and ST challenged

^a,b^Means in the same column without common superscripts differ significantly (*P* < 0.05)

### Humoral immune response

The induction of humoral immune responses against the *S.* Typhimurium-specific antigen was monitored during the weeks after ST infection to evaluate the immune-regulatory capacity of the *E. faecium* NCIMB 11181. As illustrated in Table 8, intestinal mucosa anti-*Salmonella* IgA and serum anti-ST specific IgG levels were significantly elevated (*P* < 0.05) at 7 days following ST infection in broiler chickens. *E. faecium* NCIMB 11181 addition remarkably promoted intestinal mucosa anti-*Salmonella* specific antibody production compared with those in the non-supplemented groups (*P* < 0.05). Moreover, there was significant cooperative effects on intestinal mucosa anti-*Salmonella* IgA titers between ST challenge and *E. faecium* NCIMB 11181 supplementation. Infected birds given *E. faecium* NCIMB 11181 displayed the highest anti-*Salmonella* IgA content compared with the other three groups (*P* < 0.05), infected birds alone showed higher IgA content as compared to that of the non-infected groups.

**Table 8:** Effect of dietary *Enterococcus faecium* NCIMB 11181 supplementation on anti-*Salmonella* specific IgA (OD_450nm_) of ileum mucosa and serum anti-*Salmonella* IgG (OD_450nm_) of broiler chicken challenged with *Salmonella* Typhimurium

Items	ST^1^	IgA	IgG
Dosage, mg/kg			
0	–	0.07^c^	0.45
200	–	0.12^c^	0.49
0	+	0.33^b^	1.05
200	+	0.48^a^	0.84
SEM^2^		0.015	0.066
Main factors			
0		0.20	0.75
200		0.30	0.66
Non-challenged		0.10	0.47^b^
Challenged		0.41	0.94^a^
Main factors and Interaction (*P*-value)^3^			
* Enterococcus faecium*		0.047	0.340
Challenged		0.009	< 0.001
* Enterococcus faecium* × Challenged		0.039	0.170

^1^Challenged with *Salmonella* Typhimurium, –, without ST challenged, + , with ST challenged

^2^SEM, standard error of the mean

^3^*P*-value represent the main effect of the diet, the main effect of ST challenged, and the interaction between the dietary treatments and ST challenged

^a,b^Means in the same column without common superscripts differ significantly (*P* < 0.05)

### Cecal microbiome

**Fig. 3:**
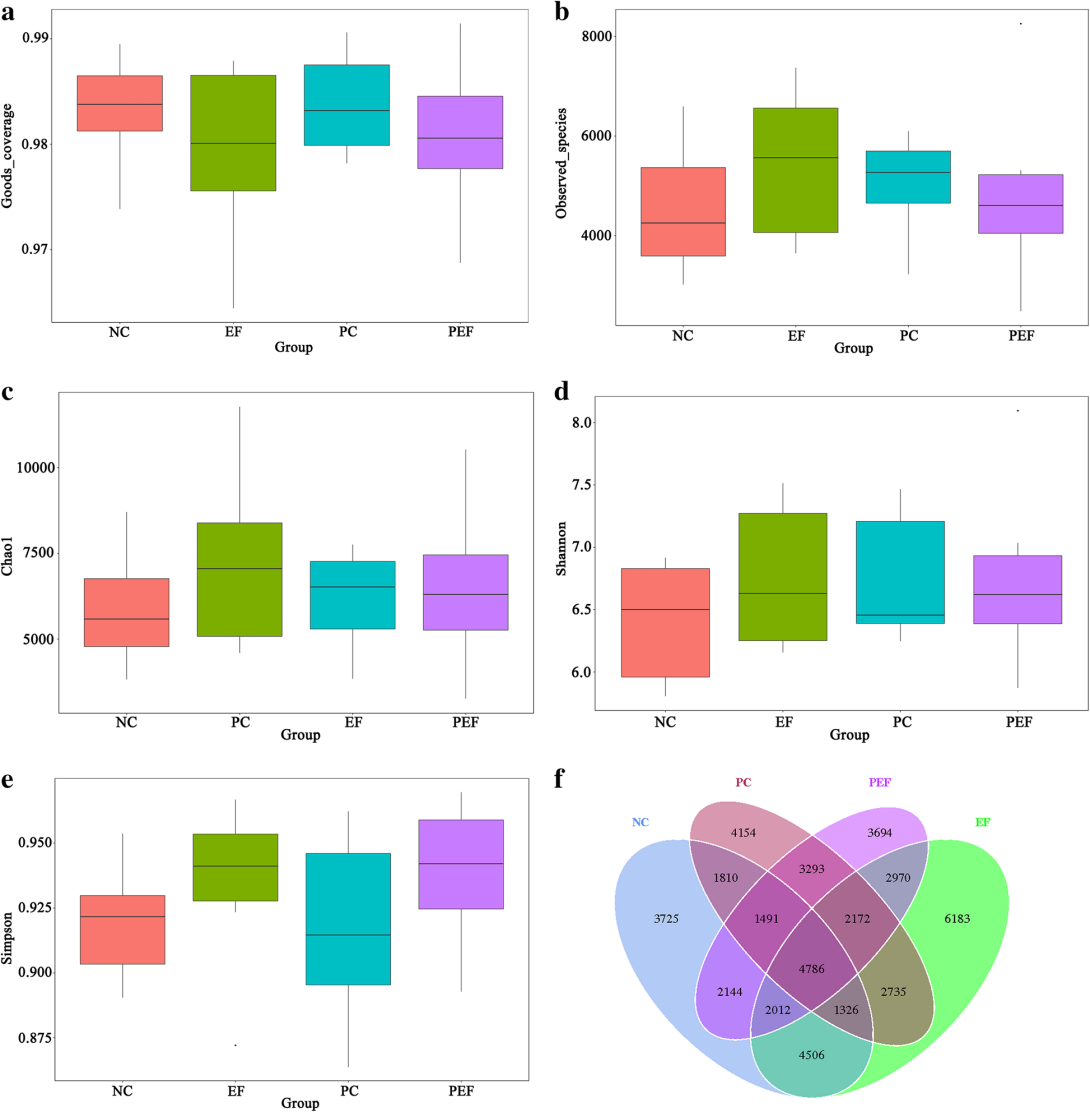
Alpha-diversity analysis of cecal microbiota communities among groups at 7 days post ST infection. **a** Goods-coverage, **b** Observed species, **c **Chao 1, **d** Shannon index, **e** Simpson index, **f** Venn diagram showing the shared OTUs by groups. NC non-infected and untreated negative control, PC ST-infected positive control without probiotics addition, EF *E. faecium*-treated group without ST infection, PEF both E. *faecium*-treated and ST-infected group. ST *Salmonella* Typhimurium

**Fig. 4:**
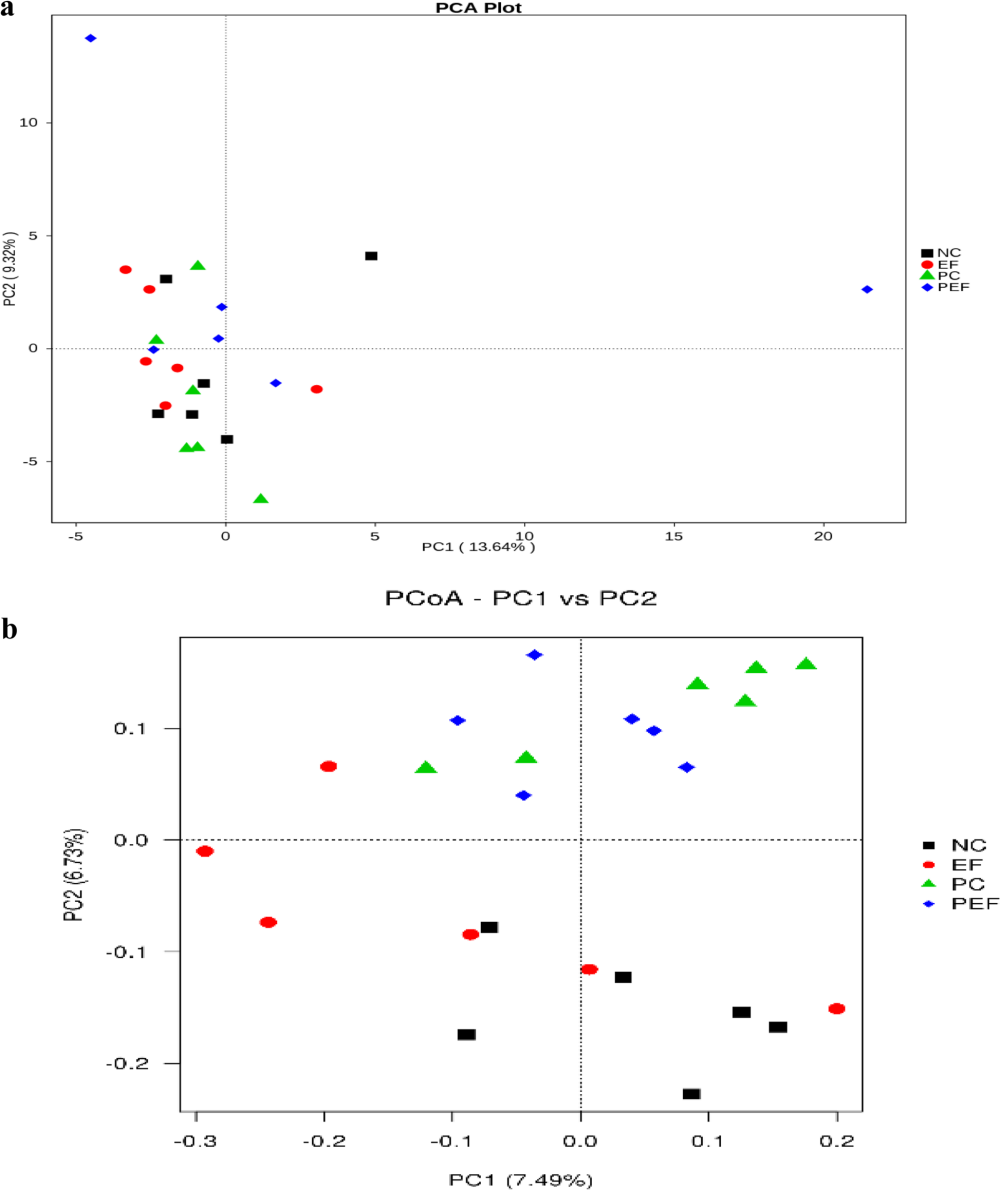
β-diversity analysis of cecal microbiota communities of broiler challenged with *Salmonella* Typhimurium (ST) at 7 days post ST infection. (**a**) Principal component analysis of the caecal microbiota based on weighted Unifrac distance (PCA plot), (**b**) Principal co-ordinates analysis (PCoA) plot based on unweighted UniFrac distance. NC non-infected and untreated negative control, PC ST-infected positive control without probiotics addition, EF *E.* *faecium*-treated group without ST infection, PEF both *E. faecium*-treated and ST-infected group. ST *Salmonella* Typhimurium

**Fig. 5:**
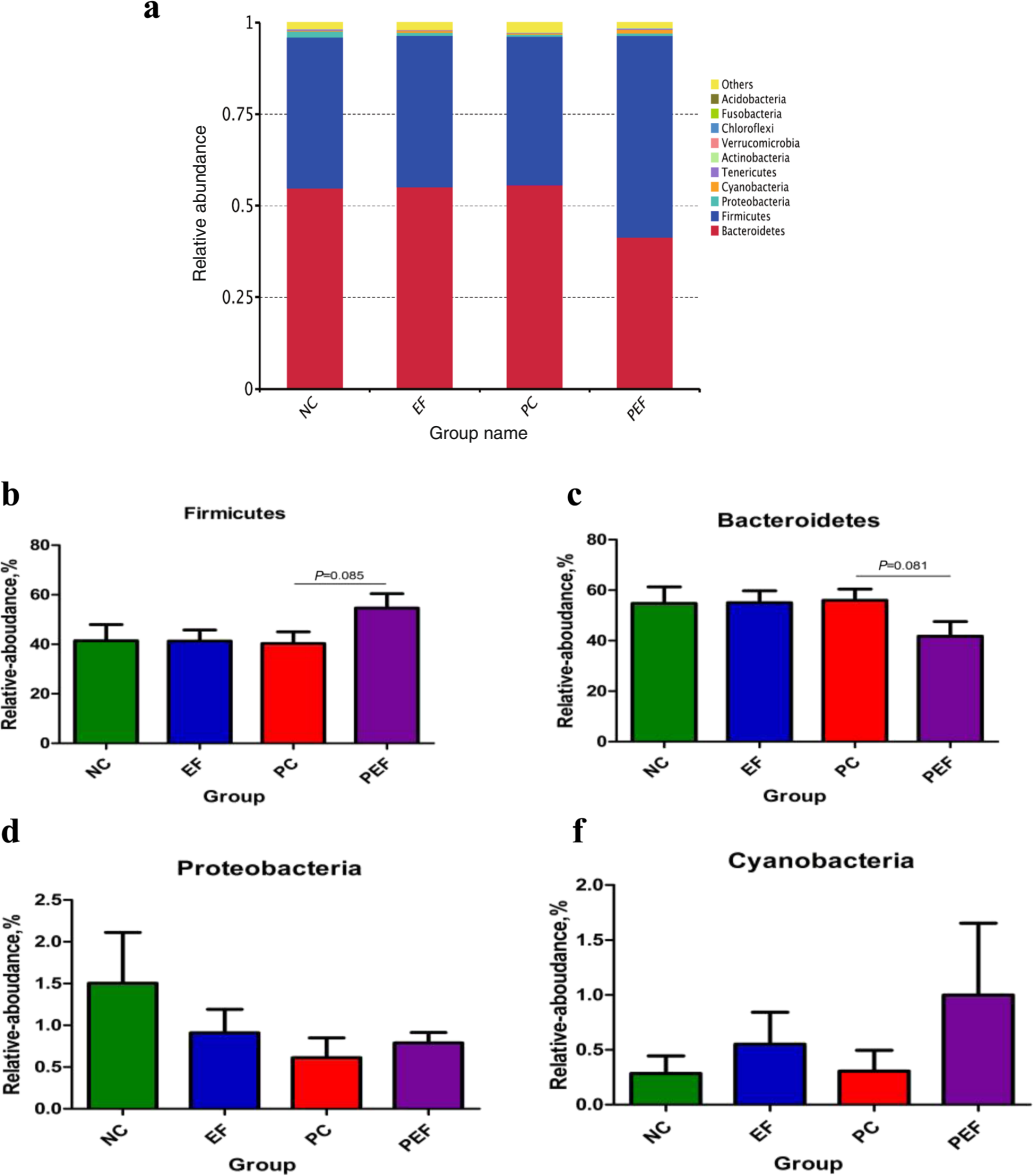
Composition of cecal microbiota of broiler chickens at the phylum level (**a**) and comparison of relative abundances of the dominant phylum within different groups (**b**, **c**, **d**, **e**). Values are presented as mean ± SEM. NC non-infected and untreated negative control, PC  ST-infected positive control without probiotics addition, EF *E.* *faecium-*treated group without ST infection, PEF both *E. faecium*-treated and ST-infected group. ST *Salmonella* Typhimurium

**Fig. 6:**
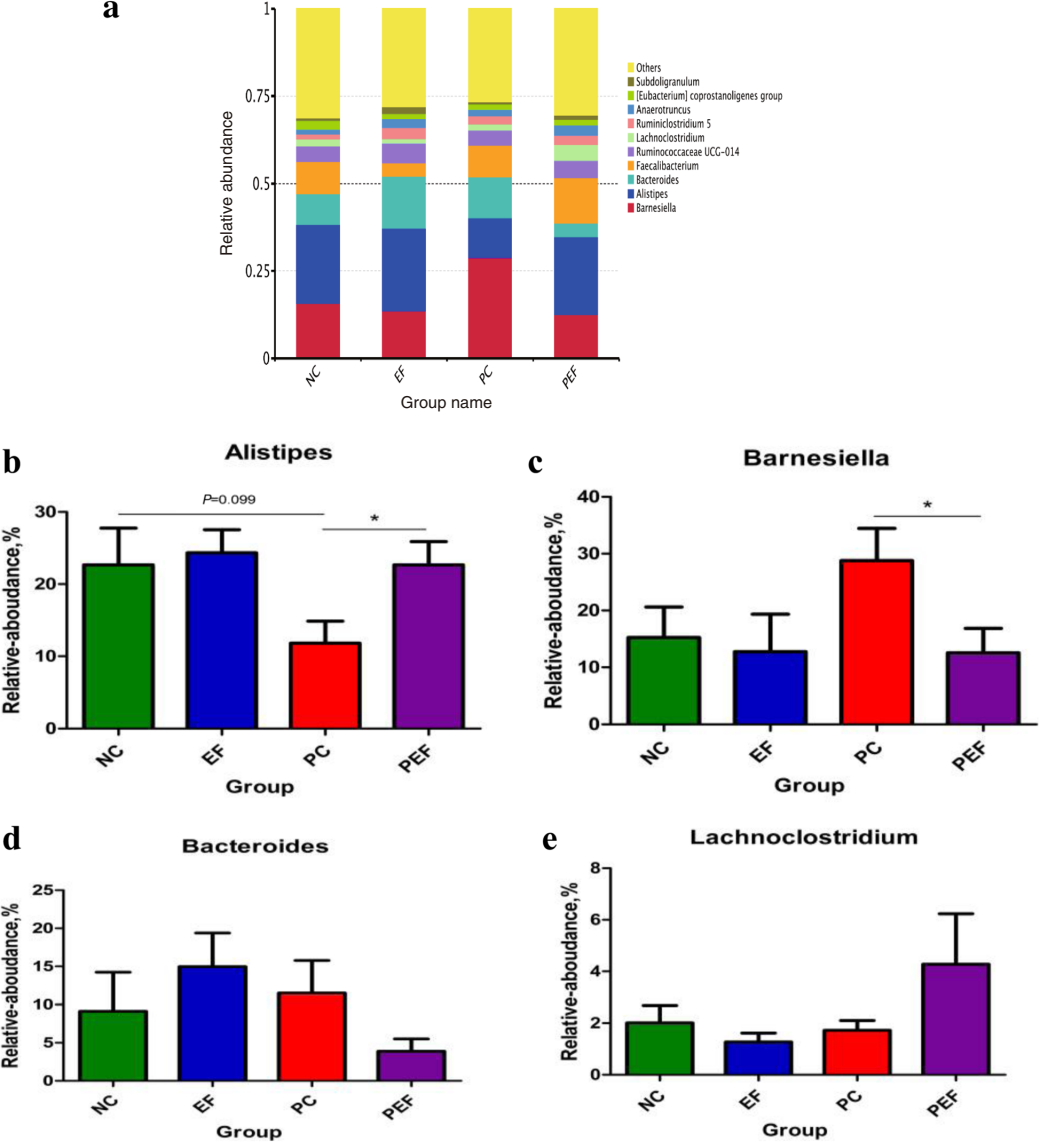
Composition of cecal microbiota of broiler chickens at the genus level (**a**) and comparison of relative abundances of the selected dominant bacterial genus in the four groups (**b, c, d, e)**. Values are presented as mean ± SEM. Asterisks (^∗^*P* < 0.05, ^ ∗∗^*P* < 0.01) indicate statistical differences between the treatment group and the pc group. NC non-infected and untreated negative control, PC ST-infected positive control without probiotics addition, EF *E.* *faecium*-treated group without ST infection, PEF both *E.* *faecium*-treated and ST-infected group. ST *Salmonella* Typhimurium

**Fig. 7:**
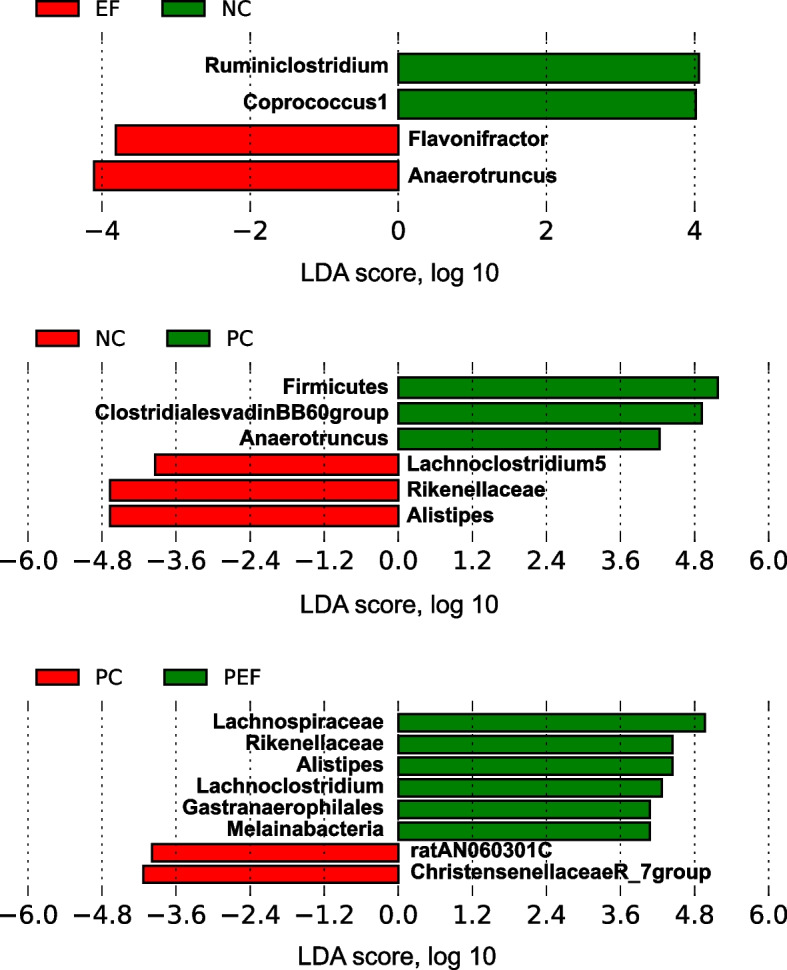
Histogram of the Linear Discriminant Analysis (LDA) score computed for differentially abundant taxa with cut-off LDA score > 2.0. NC non-infected and untreated negative control, PC ST-infected positive control without probiotics addition, EF *E.* *faecium*-treated group without ST infection, PEF both *E. faecium*-treated and ST-infected group. ST *Salmonella* Typhimurium

As shown in Table 9, a total of 542,151 high-quality sequences were obtained from 4 groups. All of OTUs were defined at 97% species similarity level, 25,765 OTUs were obtained from cecal contents samples, with an average of 1074 OTUs per sample. The species richness (observed OTUs, Chao, good-coverage) and the community diversity (Shannon, Simpson) was not influenced by ST challenge, *E. faecium* NCIMB 11181 supplementation or the interaction between *E. faecium* NCIMB 11181 and ST challenge (Fig. 3a–e). Venn diagram (Fig. 3f) indicated 4786 common core OTUs were shared among all groups, while 17,014, 21,904, 16,981, and 17,776 OTUs were unique to groups NC, PC, EF and PEF, respectively. PCA and PCoA was to be visualized β-diversity (Fig. 4a–b). Results showed that there was no obvious clustering tree associated with dietary treatments or *S.* Typhimurium infection. Conversely, the infected birds fed *E. faecium* NCIMB 11181 form a unique cluster separated from all other three groups, especially separated from the single ST-infected birds. The single ST-infected birds displayed little similarity with the other three groups (0 < *R* = 0.3704 < 1; *P* = 0.011). However, no distinct separation for cecal microbiota was found between the EF and NC groups. Differences in microbial community abundance at the phylum level between all groups were shown in Fig. 5. At phylum level, the Top 5 dominant phylum includes Firmicute, Bacteriodetes, Cyanobacteria, Proteobacteria and Tenericutes. Higher abundance of Firmicutes (*P* = 0.085) and lower abundance of Bacteriodetes (*P* = 0.061) were detected in PEF compared to the PC control. The top 10 microbes at the genus level (Fig. 6) were *Alistipes*, *Barnesiella*, *Bacterioides*, *Lachonclostridum*, *Faecalibacterium*, *Ruminococcaceae-UCG-014, Ruminiclostridium-5, Ruminococcaceae-UCG-005, Phascolarctobacterium* and *Subdoligranulum.* The abundance of genus *Alistipes* at 7 days after ST infection was notably decreased, while remarkably was increased by *E. faecium* NCIMB 11181 addition compared with the PC control (*P* < 0.05). The infected birds treated with *E. faecium* NCIMB 11181 showed an increased trend for *Lachnoclostridum*, while reduced the percentage of the genus *Barnesiella* (*P* < 0.05) and display a reduced trend in the abundance of *Bacterioides* (0.05 < *P* < 0.1). LEfSe analysis (Fig. 7) highlighted that the infected birds fed *E. faecium* NCIMB 11181 showed higher abundance in the genus *Lachnospiracease*, genus *Alistipes*, Rikenellaceae family and *Lachonclostridum*, which was similar to the changes of intestinal microbial communities of the non-infected control compared with the single ST-infected control. Under non-infected conditions, *E. faecium* NCIMB 11181 addition enriched cecal *Anaerotruncus* and *Flavonifractor* abundance as compared to the NC group.

**Table 9:** Effect of dietary *Enterococcus faecium* NCIMB 11181 supplementation on α-diversity of cecal microbiota of broiler chickens challenged with *Salmonella* Typhimurium

Items	ST^1^	Clean reads/tags	Effective reads	OTUs	Chao1	Goods-coverage	Shannon	Simpson
Dosage, mg/kg								
0	–	141,844	130,059	5982	5894.15	0.98	6.41	0.92
200	–	157,548	139,395	7020	7289.19	0.98	6.76	0.93
0	+	139,543	125,817	6338	6173.16	0.98	6.74	0.92
200	+	159,097	146,880	6425	6524.50	0.98	6.76	0.94
SEM^2^		20,771	17,075	1864	429.555	0.001	0.121	0.006
Main factors								
0		140,694	127,938	6160	6033.65	0.98	6.57	0.92
200		158,318	143,138	6723	6906.84	0.98	6.76	0.94
Non-challenged		149,696	134,727	6501	6591.67	0.98	6.58	0.93
Challenged		149,320	136,349	6382	6348.83	0.98	6.75	0.93
Main factors and Interaction (*P*-value)^3^								
* Enterococcus faecium*		0.044	0.030	0.482	0.339	0.234	0.472	0.178
Challenged		0.964	0.806	0.892	0.788	0.713	0.509	0.942
* Enterococcus faecium* × Challenged		0.816	0.379	0.550	0.565	0.898	0.514	0.775

^1^Challenged with *Salmonella* Typhimurium; –, without ST challenged; + , with ST challenged

^2^SEM, standard error of the mean

^3^*P*-value represent the main effect of the diet, the main effect of ST challenged, and the interaction between the dietary treatments and ST challenged

### Predicting the function of intestinal bacteria based on 16S rDNA data

**Fig. 8:**
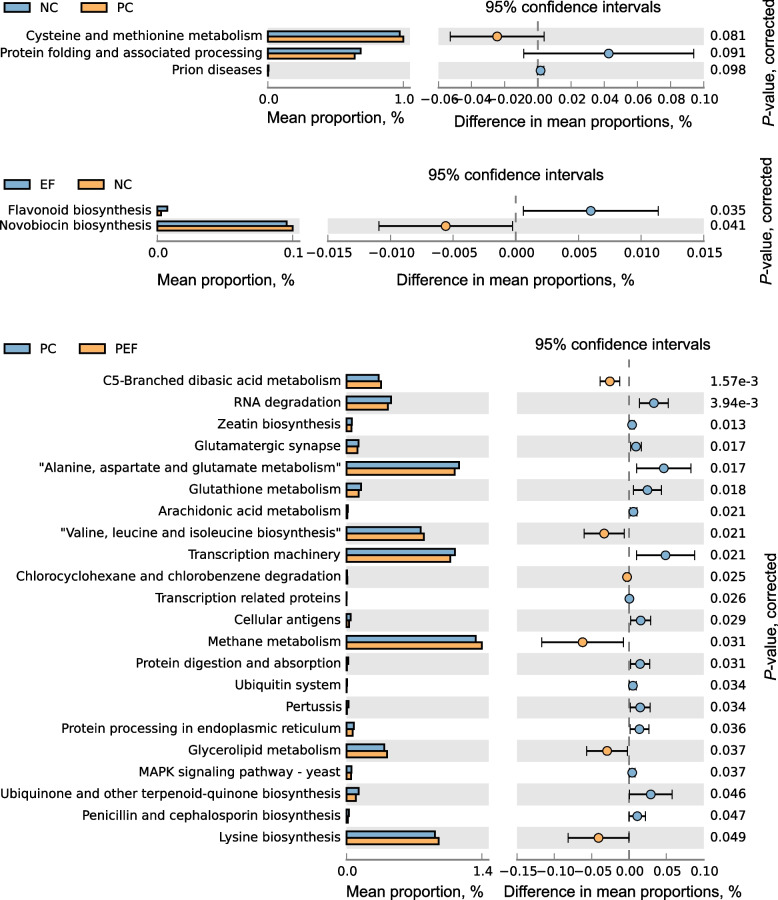
PICRUSt metagenome inference analysis (level 2) based on 16S rRNA dataset. Mean proportion of functional pathways is illustrated with bar plots and dot plots indicate the differences in mean proportions between two groups based on *P*-values obtained from two-sided Welch’s *t*-test. NC non-infected and untreated negative control, PC ST-infected positive control without probiotics addition, EF *E.* *faecium*-treated group without ST infection, PEF both *E. faecium*-treated and ST-infected group. ST *Salmonella* Typhimurium

As presented in Fig. 8, STAMP analysis revealed that pretreatment with *E. faecium* NCIMB 11181 enriched the abundance of functional genes related to flavanoid biosynthesis pathway (*P* = 0.035) but suppressed novobiocin pathway (*P* = 0.041) of cecal microbiota of the uninfected birds (at KEGG level 2). Compared with the infected chickens, infected birds given *E. faecium* NCIMB 11181 had greater numbers of functional genes involved in C5-branched dibasic acid metabolism; valine, leucine and isoleucine biosynthesis; methane metabolism; and glycerolipid metabolism and lysine biosynthesis (*P* < 0.05). While alanine, asparate and glutamate metabolism; RNA degradation, transcription machinery; MAPK signal pathway-yeast; ubiquine and other terpenoid-quinore biosynthesis; protein processing in endoplasmic reticulum; as well as glutathione metabolism (*P* < 0.05) of the cecal microbiota were suppressed in the infected birds received *E. faecium* NCIMB 11181. Thus, dietary supplementation with *E. faecium* NCIMB 11181 affected important predicted functions of the intestinal microbiota of the *S.* Typhimurium-infected birds.

## Discussion

The current study investigated whether dietary *E. faecium* NCIMB 11181 addition could be helpful for controlling *S.* Typhimurium infection and protecting intestinal health in broiler chickens. Our results showed that *S.* Typhimurium challenge caused a significant negative effect on broiler growth performance, which was similar to previous findings [5, 52, 53]. However, feeding *E. faecium* NCIMB 11181 had no remarkable growth-improving influences on chicken growth performance regardless of *S.* Typhimurium challenge. In contrast to our findings, some previous studies have showed that appropriate dose of probiotic *Enterococcus faecium* supplementation has positively affected broiler performance by increased weight gain or decreased FCR under non-challenged rearing environments [28, 54–57]*.* Similarly, our previous results demonstrated that dietary supplementation of low dose of *E. faecium* NCIMB 11181 (50 mg/kg) remarkably improved growth performance, and high dose of *E. faecium* NCIMB 11181 (200 mg/kg) notably enhanced immunity of broiler chickens under non-challenged conditions [37]. Additionally, Cao et al. [35] reported that *E. coli* K88-infected broiler chickens fed *Enterococcus faecium* showed increased growth performance, improved intestinal morphology and cecal microflora. Furthermore, our research group had reported that pretreatment with *E. faecium* NCIMB 11181 could alleviate the growth suppression caused by *Eimeria* spp./*Clostridium perfringens* co-challenge in broilers [38] and *E.coli* O78-challenged birds [39]. The inconsistent results in growth performance was possible attribute to the difference in biological features and properties of probiotics strain *Enterococcus faecium*, additive amount of *E. faecium*, feeding schedules, age of broiler chickens, challenged or not, as well as type of pathogens used for infection. In view of results of growth performance in this study, we suggested that dietary *E. faecium* NCIMB 11181 addition (200 mg/kg) did not mitigate the adverse effects of *Salmonella* Typhimurium on broiler growth performance.

Intestinal morphology (villus height, crypt depth and the V/C ratio), lesion scores, histopathological grades, bacterial colonization and translocation together with intestinal cell proliferative and apoptosis indices are important indicators of intestinal health, mucosal barrier function, integrity, permeability and recovery [58]. Additionally, intestinal epithelial cells apical junctional proteins including Claudins, Occludins, ZOs, junctional adhesion molecules and E-cadherins, also play a vital role in regulating intestinal permeability and maintaining gut barrier integrity, defense pathogens infection and inflammation response [59]. In this study, *S.* Typhimurium infection caused intestinal inflammation and gut wall impairment, as evidenced by the infiltration of inflammatory cells, shorter villus height, reduced V/C ratio, and increased TUNEL-positive cell numbers in the ileum. Also, *S.* Typhimurium infection damaged intestinal barrier function, as indicated by higher *Salmonella* load in cecal content and liver, together with downregulated tight junction *Claudin-1, Occludin*, and *ZO-1* mRNA levels and upregulated *MLCK* mRNA level in the ileum. These observation was partially in consistent with the results of previous studies [5, 52, 53, 60] in chickens, suggesting that *S.* Typhimurium infections induced the damage of intestinal morphology, promoted villus cells apoptosis, compromised the intestinal barrier integrity, and increased gut permeability of broiler chickens, thereby bacterial translocation. However, these changes caused by *S.* Typhimurium were partially alleviated by dietary inclusion of *E. faecium* NCIMB 11181, indicating that *E. faecium* NCIMB 11181 addition seem to mildly mitigate gut barrier injury induced by *S.* Typhimurium infection through improving gut morphological structure, decreasing intestinal cells apoptosis and inflammatory cells infiltration. Consistent with our findings, *Enterococcus faecium* addition not only mitigated gut injury caused by *Clostridium perfringens* [38] and *Escherichia. coli* O78 infection [35, 39], but also attenuated intestinal inflammation or gut barrier injury in chicks challenged with *Salmonella* Enteritidis [31–34] and *Salmonella* Typhimurium [60], resulting in preventing *Salmonella* infection in chickens. Based on our findings, we suggested that the addition of the probiotic product *E. faecium* NCIMB 11181 might be moderate in controlling *Salmonella* Typhimurium infection in broiler chickens. This protective and anti-*Salmonella* action induced by probiotics *Enterococcus faecium* may be attributed to its producing antimicrobial substances such as organic acids, bacteriocin and hydrogen peroxide, and adhesion inhibitors [9, 10, 61].

Intestinal inflammation will lead to destruction of intestinal tight junction and epithelial integrity. Decreased expression of tight junction proteins will aggravate intestinal inflammation. To elucidate why dietary probiotic *E. faecium* NCIMB 11181 addition could attenuate intestinal barrier impairment induced by *S.* Typhimurium, we further evaluated the changes in the intestinal mucosal immune and humoral immune responses in ST-infected broiler chickens. *TLR*-mediated signaling pathways are involved in regulating intestinal mucosal immune defense and epithelial barrier integrity as well as maintaining maintain mucosal and commensal homeostasis [62]. Pro-inflammatory cytokines were reported to increase intestinal permeability and tissue damage via the dysregulation of tight junction proteins [63, 64], while anti-inflammatory cytokines (*TGF-β, IL-4* and *IL-10*), growth factors (*EGF, GLP-2* and *IGF-2*) have been demonstrated to protect intestinal barrier function by regulating tight junction expression and facilitating the repair of damaged gut tissue [65]. In the current study, infection with *S.* Typhimurium upregulating ileal *TLR4*, *MyD88, NF-κB, IFN-γ, TNF-α, pIgR*, and the negative regulators (*Tollip* and *PI3K*) mRNA levels at the early stage of infection; which was in similar with observations of previous studies in chickens infected with *S.* Typhimurium [4, 5, 53, 66–71]. Meanwhile, intestinal mucosa anti-ST IgA and serum anti-ST specific IgG levels were also significantly elevated following ST infection in broiler chickens, which was in consistent with previous results [3, 5, 69, 70]. These observations showed that *S.* Typhimurium infection triggered intestinal local inflammation, thereby causing the disruption of intestinal barrier and the increase of gut permeability, resulting in *Salmonella* translocation and systemic inflammatory response. Interestingly, the present study found that *E. faecium* NCIMB 11181 addition only upregulated *pIgR* and *Tollip* mRNA expression level, but did not alter other genes expression profiles in the ileum of TLR-related signal pathway regardless of *S.* Typhimurium infection. On the contrary, results from previous studies have demonstrated that inclusion of *E*. *faecium* in the diet remarkably altered the genes expression profiles of intestinal *TLR*-mediated signal pathway when subjected to *S.* Enteritidis challenge in chickens [31–33]. The discrepancy of these findings might be associated with strains and administration dose of probiotics *E*. *faecium*; strains type and virulence of challenged *Salmonella*, sampling time-point and sampled tissues. *Tollip* is a negative modulator which can suppress activation of *TLR*-related signal pathway. Increased *Tollip* expression in the ileum of the chickens treated with *E. faecium* NCIMB 11181 indicated that *E. faecium* NCIMB 11181 seem to have the capability to inhibit the over-activation of *TLR* signal pathway. In addition, administration of *E. faecium* NCIMB 11181 promoted *Salmonella*-specific IgA production in intestinal mucosa of *Salmonella*-infected broiler chickens. Secretory IgA (sIgA) and its transcytosis receptor, polymeric immunoglobulin receptor (*pIgR*), along with mucus form the first lines of intestinal mucosal defenses, mainly defensing or neutralizing pathogenic bacteria and enteric toxins [72]. In this study, increased intestinal *pIgR* expression here may mean more mucosal secretory IgA antibody production in the gut, which help in reducing cecal *Salmonella* load and facilitating *Salmonella* elimination from the gut lumen during the recovery phase of infection. Higher levels of ileal sIgA together with lower *Salmonella* burden in the intestine and liver of the infected chickens fed *E. faecium* NCIMB 11181, showing that feeding *E. faecium* NCIMB 11181 had ability to provide protection against *Salmonella* infection through enhancing specific sIgA production. The findings further showed that pretreatment with probiotic *E. faecium* NCIMB 11181 could mildly alleviate *S.* Typhimurium-induced intestinal injury in chickens, possibly associated with promoting the production of sIgA in the gut.

The chicken gastrointestinal tract is colonized by trillions of microorganisms, constituting a dynamic ecosystem with significant impacts on host metabolism, productivity, immune responses and health status including gut health. Consequently, modulation of the gut microbiota and modification of the intestinal microenvironment could assist in preventing animal colonization by the pathogen [73]. More importantly, changes in gut microbe populations may be closely related to the degree of intestinal inflammation and disease resistance, which is one of the characteristics of *S.* Typhimurium infection [1, 73]. In the current study, our results revealed that neither *S.* Typhimurium infection nor dietary probiotics *E. faecium* NCIMB 11181 treatments significantly altered α-diversity of chicken caecal microbiota, indicated that the caecal microbiota diversity remained relatively stable. Nevertheless, *S.* Typhimurium infection significantly modified the indigenous microbiota composition and relative abundance of some bacterial species in the cecum of chickens, as showed by increasing relative abundance of *Barnesiella,* whereas decreasing *Alistipes* abundances, which was similar to results of Azcarate-Peril et al. [1]. In similar to our findings, *S.* Enteritidis infection also disturbed microbial composition of gut microbiota of chickens, as evidenced by expanding relative abundance of potential harmful bacteria such as *Enterobacteriaceae*, whereas decreasing potential beneficial bacteria including (i.e., butyrate-producing bacterira *Lachnospiracease*, *Bifidobacterium* and *Lactobacillus*) abundances [74]. Thus, our findings showed that *S.* Typhimurium infection disrupted microbial composition of gut microbiota of chickens, besides impairment in intestinal barrier structure. Additionally, taxonomic analysis showed that *E. faecium* NCIMB 11181 addition enriched the relative abundance of the phylum Firmicutes and the genera *Alistipes* while suppressed the relative population of the genera *Barnesiella* of the cecal microbiota of the infected birds when comparing with the single *S.* Typhimurium infected control. LEFsE analysis also indicated that the infected birds received *E. faecium* NCIMB 11181 showed higher abundance in the genus *Lachnospiracease*, *Alistipes*, Rikenellaceae family and *Lachonclostridum*, which was similar to the changes of intestinal microbial communities of the non-infected control compared with the single ST-infected control. Our study demonstrated that *E. faecium* NCIBM 11181 administration modified the structure of the gut microbiome of the *Salmonella*-infected chickens. *Alistipes*, a sub-branch genus of the Bacteroidetes phylum, exhibited protective effects against some diseases, including liver fibrosis, inflammatory colitis, cancer immunotherapy, and cardiovascular disease [75]. Butyrate-producing *Lachnospiraceae* positively correlated with good FCR performance and gut health [76], and an increase in its abundance has shown to limit expansion of aerobic enteric pathogens, reduce inflammatory diseases and prevent gut barrier dysfunction in systemic chronic lower-grade inflammation mice [77]. The genus *Barnesiella* also was reported to negatively be linked with anti-inflammatory responses but strongly correlated with proinflammatory responses in chickens [78]. Thus, higher proportion of *Lachnospiracease*, *Alistipes* and lower abundance of *Barnesiella*, accompanied by reduced *Salmonella* carrier in the cecum of *Salmoenlla*-challenged broiler chickens following *E. faecium* NCIMB 11181 administration, suggesting that pretreatment with probiotics *E. faecium* NCIMB 11181 could control *Salmonella* infection via improving gut microbiome. These data also indicated that the barrier-protecting effects of *E. faecium* NCIMB 11181 is possibly associated with the improvement of intestinal microbiome.

PICRUSt analysis revealed that functional genes related to C5-branched dibasic acid metabolism; valine, leucine and isoleucine biosynthesis; methane metabolism; glycerolipid metabolism and lysine biosynthesis were enriched; whereas functional genes involved in alanine, asparate and glutamate metabolism; RNA degradation, transcription machinery; MAPK signal pathway-yeast; ubiquine and other terpenoid-quinore biosynthesis, protein processing in endoplasmic reticulum; as well as glutathione metabolism were depleted in the cecum of the *Salmonella*-infected chickens given *E. faecium* NCIMB 11181. The findings indicated that probiotics *E.* *faecium* NCIMB 11181 administration altered functional changes of intestinal microbiota induced by *S.* Typhimurium infection. Amino acids supply was associated with energy supply, immune regulation and damage repair of gut cells, especially under challenge conditions [79]. *Salmonella* infection induced up-regulation of glycolytic process and the catabolism of amino acids at the middle and later of infection, resulting in exhaustion of energy and amino acids in chickens [80]. Such increase in amino acids biosynthesis and C5-branched dibasic acid metabolism might suggest that feeding probiotics *E. faecium* NCIMB 11181 to *Salmonella-*infected chickens potentially promoted amino acids biosynthesis processes of intestinal microbe, thereby contributing to energy supply of gut cells and repair of gut barrier impairment as well as dampening of *Salmonella*-induced intestinal inflammatory responses in broiler chickens. Asparate and glutamate metabolism, glutathione metabolism and ubiquinone biosynthesis, protein processing in endoplasmic reticulum, and other terpenoid-quinore biosynthesis reported to be involved in host nucleotide synthesis, energy metabolism of mitochondria, and redox status. Over-activation of these pathways meant that host was being exposed to stress stimulus and in a state of the imbalance of redox, resulting in oxidative stress. Suppressed pathways of functional genes of gut microbiota obtained in infected chickens after feeding *E. faecium* NCIMB 11181 indicated that *E. faecium* NCIMB 11181 pretreatment could lighten oxidative stress induced by *Salmonella* infection. We further identified that inhibition of MAPK signal pathway-yeast in the infected birds after feeding *E. faecium* NCIMB 11181, meant that *E. faecium* NCIMB 11181 could prevent *Salmonella*-induced intestinal inflammation. Hence, the increased amino acids biosynthesis, and the decreased MAPK signal pathway and redox pathway suggested that *E. faecium* NCIBM 11181 administration might play a role in alleviating *Salmonella*-induced intestinal inflammation by regulating gut microbiome, which in turn affects amino acids biosynthesis, redox pathway metabolism and MAPK signal pathway. Further experiments would be essential to confirm this possibility.

## Conclusion

In summary, *Salmonella* Typhimurium infection disrupted the balance of gut microbiota, induced intestinal inflammation and downregulated tight junction proteins genes expression, resulting in gut barrier injury and bacterial translocation in broiler chickens. Nevertheless, continuous feeding *Enterococcus faecium* NCIMB 11181 appear to alleviate *Salmonella* Typhimurium-induced gut injury mildly through modulating gut microbiota composition, promoting intestinal specific anti-*Salmonella* IgA production, along with inhibiting intestinal cells apoptosis. The results provide new information on the critical role played by dietary *Enterococcus faecium* NCIBM 11181 in controlling *Salmonella* infection in broiler chickens.

## Data Availability

The datasets used and/or analysed during the current study are available from the corresponding author on reasonable request.

## Abbreviations

A20: Protein A20
BW: Body weight
BWG: Body weight gain
CD: Crypt depth
CFU: Colony-forming unit
*E. coli*: *Escherichia coli*
*E. faecium*: *Enterococcus faecium*
EGF: Epidermal growth factor
FCR: Feed conversion ratio
GAPDH: Glyceraldehyde-3-phosphate dehydrogenase
GLP-2: Glucagon-like peptide-2
HE: Hematoxylin–eosin
HFD: High fat diet
IFN-γ: Interferon- γ
IGF-2: Insulin-like growth factor-2
IL: Interleukin
IOD: Integral optical density
KEGG: Kyoto Encyclopedia of Genes and Genomes
LDA: Linear discriminant analysis (LDA)
LEfSe: Linear discriminant analysis combined effect size measurements (LEfSe)
MAPK: Mitogen-activated protein kinase
MLCK: Myosin light chain kinase
MyD88: Myeloid differential protein-88
NF-κB: Nuclear factor kappalight-chain-enhancer of activated B cells
NRC: National Research Council
PCoA: Principal coordinate analysis
PCR: Polymerase chain reaction
PIgR: Polymeric immunoglobulin receptor
PI3K: Phosphatidylinositol 3-kinase
PLS-DA: Partial least squares discriminant analysis
SIgA: Secretory immunoglobulin A
TGF-β4: Transforming growth factor beta 3
TLR: Toll-like receptor
TNFSF15: Tumor necrosis factor superfamily 15
Tollip: Toll-interacting protein
TUNEL: Terminal-deoxynucleoitidyl transferase mediated nick end labeling
VH: Villous height
VH:CD ratio: The ratio of villus height to crypt depth
ZO-1: Zonula occludens-1
